# Cell Motility Dynamics: A Novel Segmentation Algorithm to Quantify Multi-Cellular Bright Field Microscopy Images

**DOI:** 10.1371/journal.pone.0027593

**Published:** 2011-11-09

**Authors:** Assaf Zaritsky, Sari Natan, Judith Horev, Inbal Hecht, Lior Wolf, Eshel Ben-Jacob, Ilan Tsarfaty

**Affiliations:** 1 Blavatnik School of Computer Science, Tel Aviv University, Tel Aviv, Israel; 2 Department of Clinical Microbiology and Immunology, Sackler School of Medicine, Tel Aviv University, Tel Aviv, Israel; 3 School of Physics and Astronomy, Tel Aviv University, Tel Aviv, Israel; University of Milano-Bicocca, Italy

## Abstract

Confocal microscopy analysis of fluorescence and morphology is becoming the standard tool in cell biology and molecular imaging. Accurate quantification algorithms are required to enhance the understanding of different biological phenomena. We present a novel approach based on image-segmentation of *multi-cellular* regions in *bright field* images demonstrating enhanced quantitative analyses and better understanding of cell motility. We present *MultiCellSeg*, a segmentation algorithm to separate between multi-cellular and background regions for bright field images, which is based on classification of local patches within an image: a cascade of Support Vector Machines (SVMs) is applied using basic image features. Post processing includes additional classification and graph-cut segmentation to reclassify erroneous regions and refine the segmentation. This approach leads to a parameter-free and robust algorithm. Comparison to an alternative algorithm on wound healing assay images demonstrates its superiority. The proposed approach was used to evaluate common cell migration models such as wound healing and scatter assay. It was applied to quantify the acceleration effect of Hepatocyte growth factor/scatter factor (HGF/SF) on healing rate in a time lapse confocal microscopy wound healing assay and demonstrated that the healing rate is linear in both treated and untreated cells, and that HGF/SF accelerates the healing rate by approximately two-fold. A novel fully automated, accurate, zero-parameters method to classify and score scatter-assay images was developed and demonstrated that multi-cellular texture is an excellent descriptor to measure HGF/SF-induced cell scattering. We show that exploitation of textural information from differential interference contrast (DIC) images on the multi-cellular level can prove beneficial for the analyses of wound healing and scatter assays. The proposed approach is generic and can be used alone or alongside traditional fluorescence single-cell processing to perform objective, accurate quantitative analyses for various biological applications.

## Introduction

Molecular imaging via confocal microscopy is widely used to infer cellular and molecular biological processes. Many advances have occurred in microscopic imaging such as high throughput data collection, but automatic analysis is still lagging behind. In many cases, analysis is still performed manually and is the bottleneck in visual-based cellular studies. Combining quantitative fluorescent and bright field microscopy with information on cellular morphology and texture will enhance understanding of the biological processes involved. New approaches for automatic processing and extraction of objective and accurate quantitative measures, which are exceedingly important for progress in this field, are thus sorely lacking.

A variety of software tools and imaging apparatuses exist to enable high throughput studies. Cellular morphology characteristics that decipher various biological activities, obtained via bright-field imaging modalities such as DIC, are considered hard to process and analyze and hence development of designated tools and algorithms for these microscopy categories has been neglected. Most of the existing work on bright field microscopy segmentation relies on some local texture descriptor followed by applying a threshold or global refinement [1], [2]. Other approaches manipulate the image acquisition to make the segmentation task easier [3], [4].

Much of the current microscopy-based cellular research focuses on the single-cell level. This approach relies on algorithmic framework with powerful image analysis tools (e.g., [5], [6], [7]) and requires single cell segmentation and tracking. However, direct segmentation of single cells in bright field images, especially of cells growing in dense populations, is an extremely challenging task and is prone to algorithmic errors. These errors are mainly derived from the difficulty to locally define the borders of a single cell growing in a cluster, a task that is sometimes not trivial even for an expert. We propose a specific application to analyze clusters of cells, in addition to the common fluorescent-based analyses. This approach is less susceptible to algorithmic faults and noisy data and can be performed on mass data, thus enabling a truly robust automatic analysis that is based on quantitative statistical measurements of cellular regions.

Cellular motility is a significant process in many biological systems. For example, most deaths of cancer patients do not occur due to the primary tumor but rather due to tumor cells that acquire motile-invasive phenotype and develop metastases. A well-studied model for cell motility leading to metastasis includes Met tyrosine kinase receptor and its ligand, Hepatocyte Growth Factor/Scatter Factor (HGF/SF) [8], [9]. A better understanding of the changes that occur during HGF/SF-induced motility and development of new anti-metastatic targeted therapy are considered major challenges in biomedical research.

Here, we investigate HGF/SF-induced cell motility via a novel approach that is based on Machine-learning classification to segment and analyze cellular regions in bright field images, similar to the general framework described by Shamir *et al.* [10].

### Wound Healing Assay

Wound healing assay is the gold standard method to study cell motility and migration [11], [12]. It is performed by following the closure of a wound formed by scratching a confluent cell culture. The scratch is then imaged at different times during the healing process and its area is measured. The rate of change in the wound's area is recorded and can be compared with other cells and treatments. In some studies such as Yarrow *et al.* [13], the wound healing assay is adapted to a 384 well plate, which provides mass data and allows high-quality quantitative analysis of the assay, which has not been available before. However, manual analysis becomes unfeasible when large amounts of data need to be processed.

Marking the region of interest (ROI) in the wound area for each image is the basic task required to analyze wound healing assays. Automating this process would save time and effort in future studies, especially considering the high amount of data currently available. Correct automatic wound tagging may enable high throughput analysis while enhancing the temporal sampling resolution, which is currently very limited. Several algorithms and tools have recently been proposed to deal with this task [1], [14], [15], [16] that significantly improved the ability to perform automatic analysis.

### Available Tools for Automatic Analysis of Wound Healing Assay

TScratch [1] is a freely available software that uses fast discrete curvelet transform [17] to segment and measure the area occupied by cells in an image. The curvelet transform extracts gradient information in many scales, orientations and positions in a given image, and encodes it as curvelet coefficients. TScratch selects two scale levels to fit the gradient details found in cells' contours, and generates a curvelet magnitude image by combining the two scale levels, which incorporates the details of the original image in the selected scales. Morphological operators are further applied to refine the curvelet magnitude image. As a final step, an automatic threshold is applied to partition the curvelet magnitude image into occupied and free regions. This approach was first applied for edge detection in microscopy images [18]. However, this algorithm suffers from several drawbacks: dependence on parameter settings, shortcoming in detecting smaller wound regions and insufficient robustness to different cell types or challenging imaging conditions. Additional tools suffer from incompatibility to bright field images [5], [6], [14], or employ image processing tools that are incapable of dealing with data variability [15], [16], [19], [20], as they are mainly based on quantifying edges density or simple local texture descriptors within the image.

### Cell Scattering Assay

Cell scattering is the interruption of cell-to-cell interaction that results by dispersal of cells. It is an important phenomenon in pathological, developmental and cell migration investigations. HGF/SF induces cell scattering through the tyrosine kinase-type HGF/SF receptor c-Met [21], [22], [23]. Analyses of scatter assays are almost always qualitative-based. Cell scattering is scored by an expert's manual decision, based on spreading and dispersion of epithelial colonies. Only few attempts have been made to quantify objective measures for cell scattering. Kort *et al.* [24] suggested a simple image-processing application that detects and counts clusters of cells and single cells based on fluorescent marking. Although proving high correlation with manual counting, they do not show a quantitative measure to describe cell scattering. Powell *et al.* [25] quantified scatter response of MDCK cells to HGF/SF by measuring the distances between nearest neighbor cell's nuclei, and demonstrated that addition of low concentrations of HGF/SF resulted in cell dispersion.

We propose a novel segmentation algorithm which comprises of a spatially local stage followed by a global stage, to automate the partition of a bright field image into regions of cells versus background. Based upon this algorithm, we developed a novel multi-cellular texture-based approach for DIC microscopy that enables classification and objective measurement of cell scattering.

## Materials and Methods

### Cell Lines

DA3 cells (derived from the mouse mammary adenocarcinoma cell line D1-DMBA-3, induced in BALB/C mice by dimethylbenzanthracene) [26] were grown in DMEM (Gibco ± BRL) supplemented with 10% heat-inactivated FCS (Gibco ± BRL).

Madin-Darby canine kidney epithelial cells (type 2) (MDCK) were kindly provided by Dr. K. E. Mostov (University of California San Francisco, San Francisco, CA). MDCK cells were grown in DMEM (Gibco ± BRL) supplemented with 5% heat-inactivated FCS (Gibco ± BRL).

### Wound Healing Assay

The assay was carried out with DA3 cells. When the cells reached 90% confluence, a scratch was generated using a 200 µl sterile tip. Cells were incubated in DMEM 0.1% FCS with or without HGF/SF (80 ng ml^−1^) and imaged overnight every few minutes.

### Data Sets for Evaluation of *MultiCellSeg*

To evaluate our segmentation algorithm, we used wound healing images available from TScratch website, images received with the courtesy of S. Izraeli and I. Witz (personal communication), and new images that were acquired in our lab. These 126 images were manually marked to quantify our algorithm's performance. Twenty arbitrary images from all data sets were selected to train the patches- and the regional-classifiers.

The 126 images were partitioned to the following four data sets:

- Only 24 images were available from the TScratch website. The imaging configuration is detailed in [1]: “Two crosses were scratched in each well, and these were instantly center-imaged at 5× magnification, using a Zeiss Axiovert 200 M microscope equipped with a Zeiss AxioCam MRm camera with maximum contrast (Carl Zeiss AG, Feldbach, Switzerland)”. This data set was denoted TScratch;
- 20 images of cell populations of brain metastatic melanoma were acquired in the I. Witz lab using an inverted microscope (Eclipse TE 2000-S; Nikon, Enfield Enfield, CT, USA) fitted with a digital camera (DXM1200F; Nikon). Ten of these over-produced CLDN1, the other 10, infected with a mock plasmid. This data set was denoted Melanoma;
- 28 DIC images (pixel size 0.625×0.625 µm), denoted Init, were acquired using LSM-410 microscope (Zeiss, Germany) in non-confocal mode from 2 single-well experiment in our lab: DA3 cells were plated on glassbottom 35 mm diameter microwell plates (MatTek, Ashland, MA) and imaged overnight every 3 minutes.
- A set of 54 DIC images (pixel size 1.24×1.24 µm), denoted SN15, were acquired LSM-510 microscope (Zeiss, Germany) in non-confocal mode, from 27 different wells acquired in a multi-well experiment performed in our lab: DA3 cells were grown in 24 well plates and imaged overnight every 15 minutes. The position of each scratch was predefined, and a macro that repetitively positions the microscope to each point was executed. The acquired time-lapse images were used for the analysis until occurrence of the first contact between opposing borders of the wound.

Each image was manually segmented to enable comparisons between accuracies of different segmentations.

### Scatter Assay

The assay was carried out with MDCK cells as previously described [31], [32]. Cells were seeded in 96-well plates (Corning, NY, USA) (4,000 cells in each well) and incubated overnight with or without HGF/SF (80 ng ml^−1^), examined under a microscope (CLSM-410, Zeiss, Germany) and photographed.

### The Segmentation Algorithm

The proposed algorithm is based on statistical learning of the local appearance of cellular versus background (non-cell) small image-regions (denoted *patches*) in wound healing assay images. As a classification application, it is comprised of two phases, training and testing. In the training phase, a set of (manually) tagged images are given as input to a standard classification algorithm that calculates a linear statistical model that assesses the expected appearance of a background patch, the *patch classifier*. In the testing phase, the acquired model is applied to classify new, untagged images.

**Figure 1:**
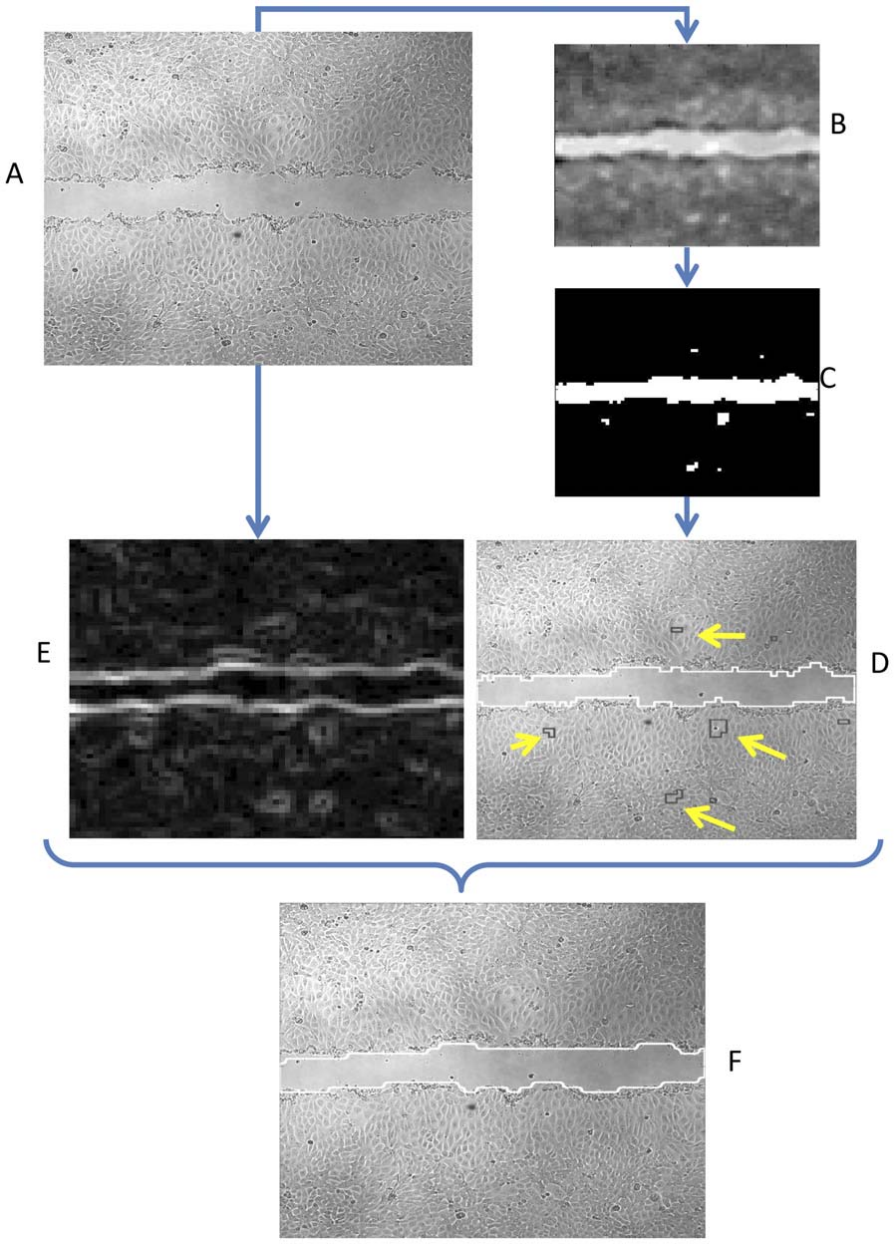
MultiCellSeg Algorithmic overview. (A) Initial image. (B) Apply classification on 20×20 pixels patches to produce confidence score: image regions with higher intensity are more likely to be non-occupied (background), darker regions are more likely to be cellular. (C) Discrete version, produced by applying an automatic threshold on the confidence image. (D) The regional classifier is applied to discard cellular regions misclassified as non-occupied: non-occupied regions contours are marked in white while filtered regions are marked in black (some are pointed with yellow arrows). The union of the black and white contours is the output of the first phase, patches classification. (E) Initial image's energy map for Graph-Cut refinement. (F) Final segmentation: result of Graph Cut segmentation using the output of the regional classifier as its baseline.

A given image (Fig. 1A) is partitioned into patches (typically of size 20×20 pixels). For every patch, the patch classifier outputs a *confidence score* that represents the model's “certainty” in that patch being “cell” or “background”, which is given by the Euclidian distance of its feature-vector representation to the hyper plane defined by the linear model. This is demonstrated in Fig. 1B, where bright pixels are more likely to be “background”, while dark are more likely to be “cellular”. This is followed by applying an automatically-selected threshold on the confidence scores to define the initial image segmentation (Fig. 1C). The next step is to apply another, separate pre-trained classifier, denoted the *region classifier*, to reclassify cell regions previously classified by the patch classifier as background. It is designed to identify spatially connected components of patches that were originally misclassified as “background” (Fig. 1D); the patches' grouping introduces a substantial advantage that enables to consider a large region in the image, which contains much more image-textural information than the local patches. Graph-cut based segmentation algorithm is used to refine the spatial classification and produce the final partitioning (Fig. 1E–F). This algorithm is denoted *MultiCellSeg* and is described in detail in File S1. Using this approach, a small amount of high quality, manually analyzed data can be used to produce large amounts of automatically annotated data of similar quality.

**Figure 2:**
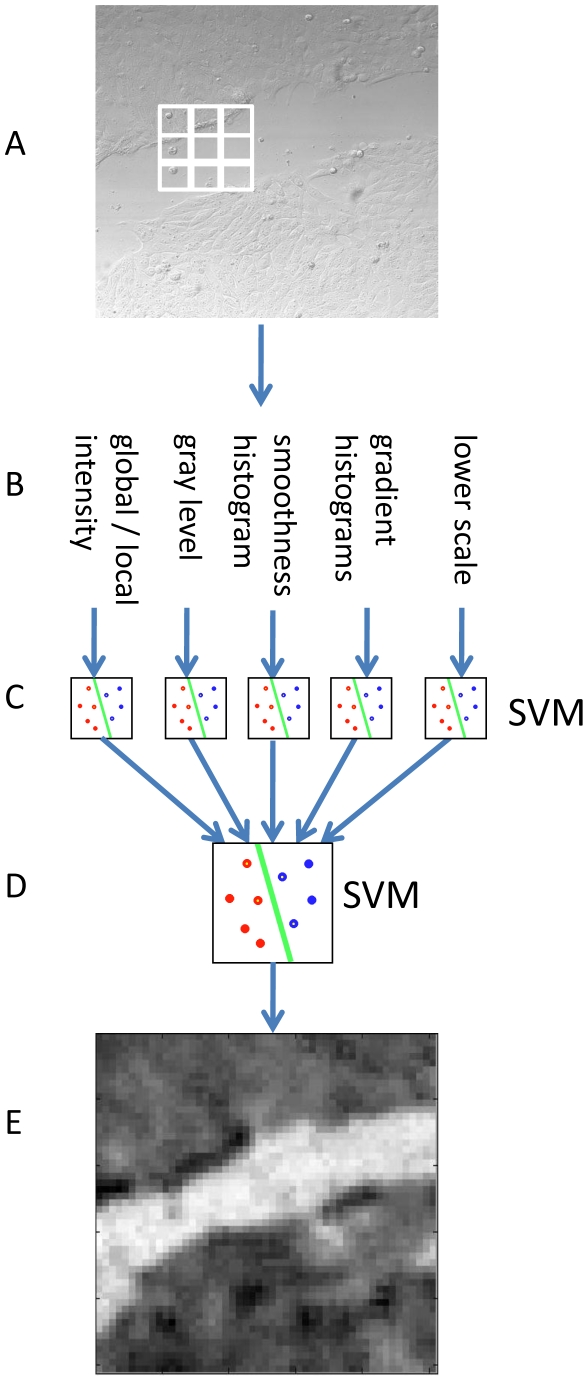
Patches classification. (A) Initial image is divided into patches (20×20 pixels per patch for the wound healing application). (B) Five sets of basic image-processing features are extracted per patch. (C) Five pre-trained Support Vector Machines (SVM) are applied to classify the feature sets. (D) A confidence score is produced for each combination of patch and features set. An additional pre-trained SVM is applied on the assembly of the confidence scores. (E) The final confidence map, brighter patches correspond to high probability of non-occupied regions.

Given a test image, the following steps are applied to define the background as the region of interest:

- Create patches' grid, extract texture-based features (such as patch's gradient histogram), apply cascade of SVMs to classify all patches as cell/background (visualized in Fig. 1C, and in more detail in Fig. 2);
- Discard cellular regions that were marked as background by the patches classifier and reclassified as cellular by the region classifier (Fig. 1D);
- Apply graph-cut and output the region of interest (Fig. 1F).

## Results

To compare the MultiCellSeg with alternative approaches, we considered the available automatic tools for wound healing analysis. Several researchers (e.g., [20], [27]) use a combination of edge-detection or simple local texture descriptors and morphological operators. These tools can be tuned to fit specific data sets but bear difficulties in handling diverse ranges of image-acquisition conditions and different cell types. CellProfiler [5] has many useful applications, but its wound healing algorithm is using generic modules that are more appropriate for other applications; its performance in segmenting wound healing images under the default settings is very poor hence direct comparison was discarded. To the best of our knowledge, the only freely available software for automatic analysis of wound healing that performs reasonably well on bright field images without specific parameter setting is TScratch [1]. The quality of MultiCellSeg was therefore compared with it.

Both MultiCellSeg as well as TScratch can be seen as composed of two parts. First, the original image is used to create a new one, in which the intensity of each pixel represents the algorithm's confidence in its classification. Then, this image is used to define the final ROI.

The first phase in TScratch is the construction of the curvelet magnitude image, whereas in our approach, it is the generation of the classifier's confidence image. The second phase in TScratch is the automatic setting of a threshold and then the application of morphological operators. In MultiCellSeg, the second phase includes removal of erroneous tagged regions and contour refinement.

Thus, the comparison of these algorithms is performed in two steps. The robustness of the first phase is measured by examining the Receiver Operating Characteristic (ROC) which plots true-positive versus false-positive classification rates of the pixels in each image across the entire range of possible thresholds of the confidence threshold, encoding the true potential of the underlying approach. The second measure is a direct comparison between the algorithms' final tagging.

**Figure 3:**
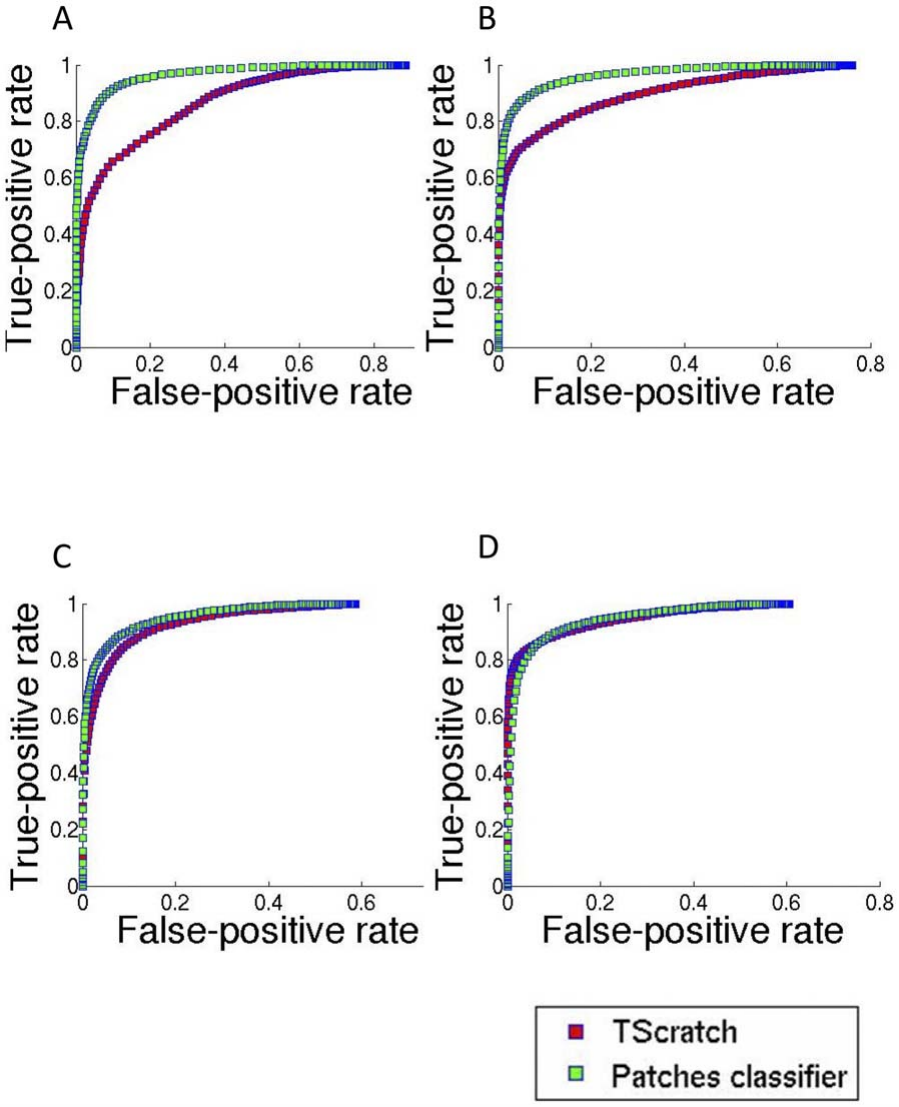
Segmentation results: ROC curves. Receiver operating characteristic (ROC) curves (red – TScratch, green – MultiCellSeg's patch classifier). The x-coordinates represent the false-positive rate; the percent of image's pixels that were classified incorrectly to background pixels out of all cellular pixels. The y-coordinates are the true-positive rate; the percent of background pixels tagged correctly out of all image's background pixels. Each curve was produced by averaging the ROC curves of all images in the data set. (A) Init: single well DA3 cells acquired at high temporal resolution (28 images), (B) SN15: multi-well DA3 cells taken at different imaging conditions (54 images), (C) Melanoma: brain metastatic melanoma cell lines (20 images), (D) TScratch: all available TScratch's sample images taken from http://www.cse-lab.ethz.ch/index.php?&option=com_content&view=article&id=363, containing cell lines with various morphologies (24 images).

The ROC curves comparing TScratch with MultiCellSeg are presented in Fig. 3. The x-coordinate represents the false-positive rate, which is the percent of pixels that were incorrectly tagged as background, out of all cellular pixels of the given image. The y-coordinate is the true-positive rate, which is the percent of background pixels correctly tagged, out of all image's background pixels. Each curve was produced by averaging the ROC curves of all images in the data set. The higher the threshold is, the lower the false positive classification rate will be, but this comes at the cost of true positives. When comparing the potential accuracy of several classification algorithms, an algorithm that has higher true-positive rate for any fixed false-positive rate values is proved to be the best. Thus, higher curves correspond to more discriminative algorithms. ROC is described in detail in File S1.

To set TScratch's threshold, MultiCellSeg's true-positive rate was used to define TScratch's threshold such that the same true-positive rate was achieved; the total error rate (rate of false-negative and false-positive pixels out of all image's pixels) was compared after applying this threshold. It is important to note that this process actually “upgraded” TScratch; using a predetermined threshold, or a dynamic threshold using Otsu's method [28] resulted with inferior performance (results not shown).

**Figure 4:**
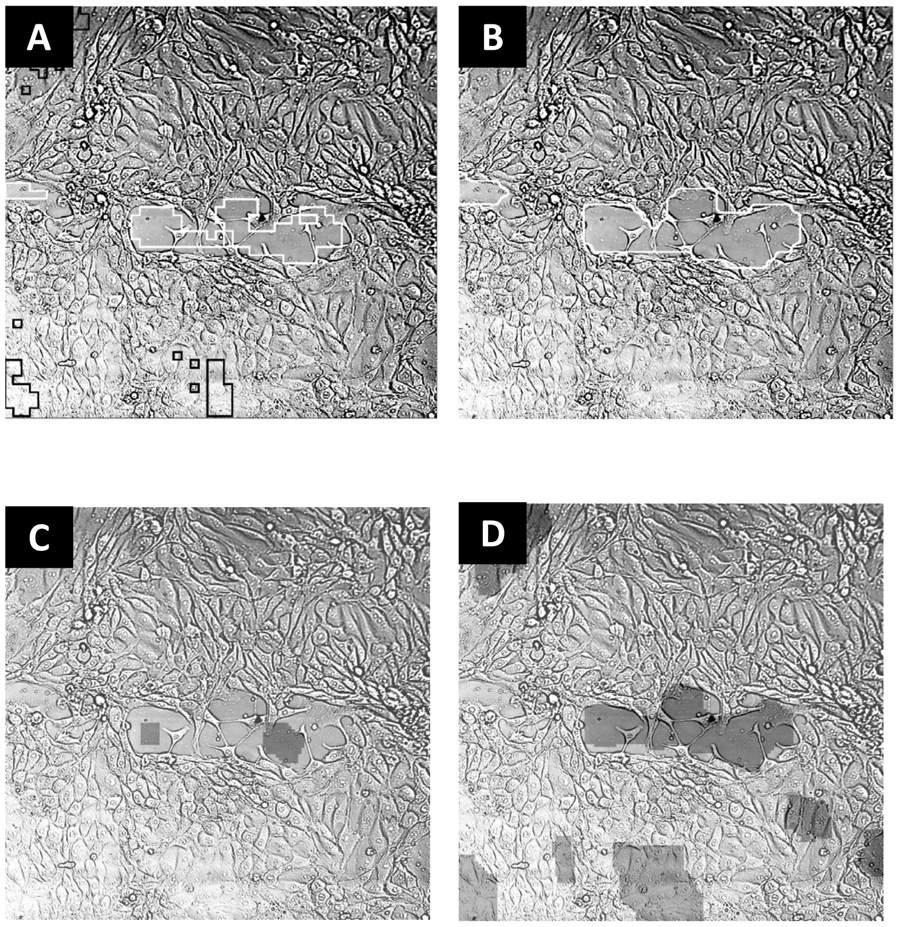
Visual segmentation comparison. Visual comparison on images with small non-occupied regions: (A) MultiCellSeg's before applying graph-cut segmentation: non-occupied regions contours are marked in white while filtered regions are marked in black. (B) After graph-cut refinement. (C) TScratch result with automatic threshold. (D) TScratch results after manual adjustment of the threshold such that most of the non-occupied regions are marked.

Table 1 compares the final segmentation results, after patch-classification, and the final segmentation (after applying region-classification and graph-cut refinement) with TScratch's performance. Each entry is an average accuracy on all images in the designated data set. MultiCellSeg surpasses TScratch (p-value 0.001 for the TScrach dataset and less than 0.000015 for the other datasets, paired Student t-test): it outperforms in 75-95% of the images for all data sets, and presents accuracy of over 3% better than TScratch's. Since TScratche's accuracy is over 90% for most datasets, our approach actually decreases the rate of misclassified zones from around 10% to around 7%, and this improvement may turn crucial. A qualitative demonstration of MultiCellSeg's superiority on images with narrow background regions is presented in Fig. 4.

**Table 1:** Segmentation results.

Dataset Name	Init	SN15	Melanoma	TScratch
Number of images	28	54	20	24
Patch Classifier Accuracy (%)	95.5	94.5	90.5	89.8
MultiCellSeg Accuracy (%)	**96.9**	**95.3**	**91.2**	**92.2**
TScratch Accuracy (%)	92.3	92.3	87.0	89.8
pValue: Patch Classifier vs. TScratch	1.9e-4	1.8e-5	4.6e-5	0.95
pValue: MultiCellSeg vs. TScratch	9.1e-8	1.38e-5	3.37e-6	0.01
Percent of Images for which MultiCellSeg Outperforms TScratch (%)	**95**	**85**	**90**	**75**

Summary of segmentation accuracy and significance. Accuracy is defined as percent of correctly tagged pixels out of the total number of pixels in all images. Accuracy was calculated for the patches classifier (intermediate segmentation) and for the final MultiCellSeg segmentation and was compared to TScratch accuracy on the same set of images. pValue calculated as a paired t-test on the accuracy sequences: patches classification vs. TScratch MultiCellSeg vs. TScratch for each image. Percent of images for which MultiCellSeg outperforms TScratches' refers to the percent of images in the dataset that are better segmented by MultiCellSeg in comparison to TScratch.

### HGF/SF Effect on Wound Healing

To validate the effect of HGF/SF on the healing rate, we trained models to analyze a specific time-lapse microscopy multi-wells experiment, where DA3 cells in some wells were treated with HGF/SF. An image was sampled every ∼75 minutes until first contact formation between cells from opposing borders of the wound. Several arbitrary images were selected and manually marked for training. Every image was segmented and the wound area was examined as a function of time. Two measures were determined in this study: the linearity of the healing process and the change in wound closure rate under HGF/SF treatments.

**Figure 5:**
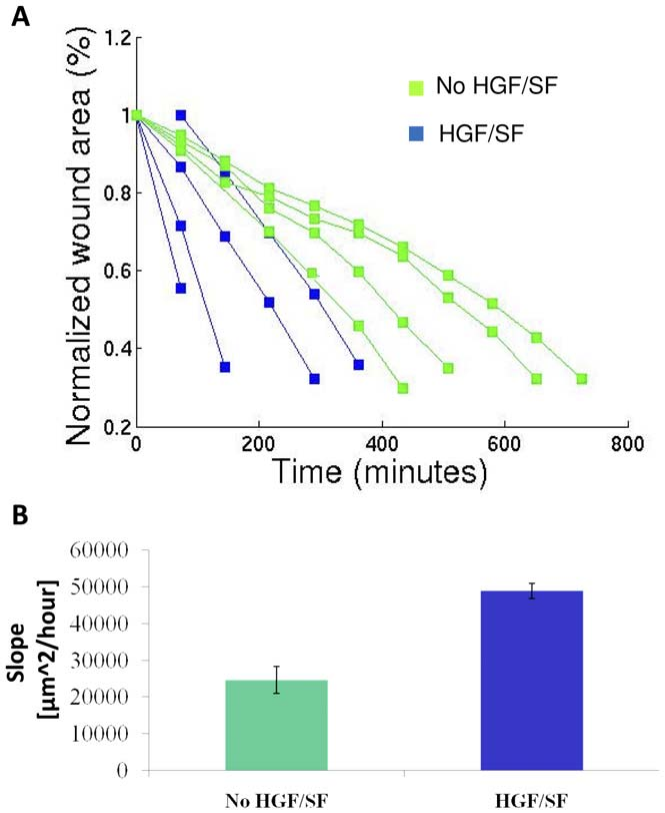
HGF/SF accelerates wound healing. (A) Normalized wound area over time: treated vs. untreated. The wounds are normalized such that each initial wound is set to 1. It is shown that the healing rate is linear (r>0.978 for all experiments, p<0.0003 via Pearson linear correlation coefficient, for all experiment with more than 3 time points). (B) Healing slope: treated vs. untreated. The slope is calculated based on the wound area change over time. It is shown that HGF/SF accelerates healing (∼2 folds, p<0.016 via Wilcoxon rank sum test).

The healing rate was linear in all experiments (r>0.978, p<0.0003 via Pearson's linear correlation coefficient, for all experiments with more than 3 time points), as is visualized by the normalized wound area over time (Fig. 5A). The healing slope is calculated based on rate of change in wound area. HGF/SF accelerates healing about 2-fold (p<0.0016 via Wilcoxon rank sum test, which has no prior assumptions on the data distribution, thus it is a strict test) (Fig. 5B).

### Objective Measure for Cell Scattering

**Figure 6:**
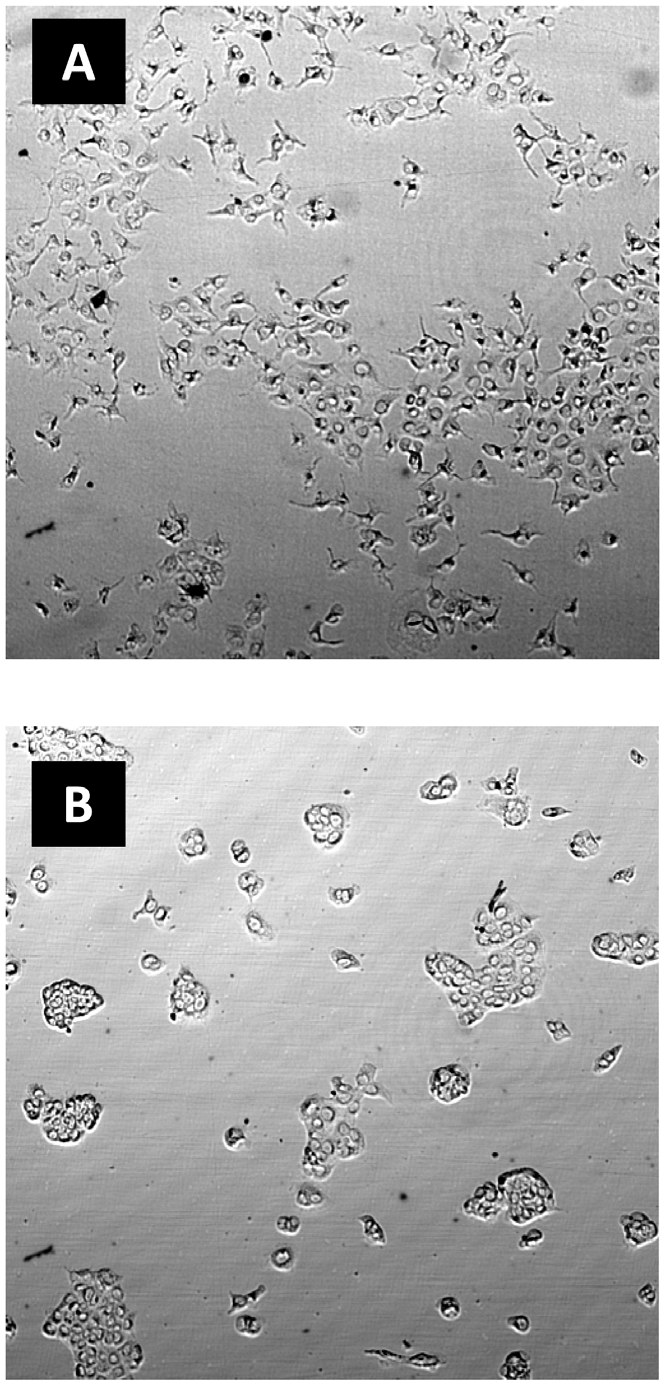
Scattered/none scattered examples. (A) Scattered image. (B) None scattered image.

Each cell scattering image was visually classified by an expert as “scattered” (10 images) or “none scattered” (22 images) and was verified by 3 independent experts. An example of “scattered” and “none scattered” images is illustrated in Fig. 6.

Every image was described by a feature vector of size 10 (as detailed below). We used “leave one out” to objectively evaluate the cell scattering measure; for each image, an SVM was trained on the remaining 31 images, and the left-out image was used to evaluate the trained model.

**Figure 7:**
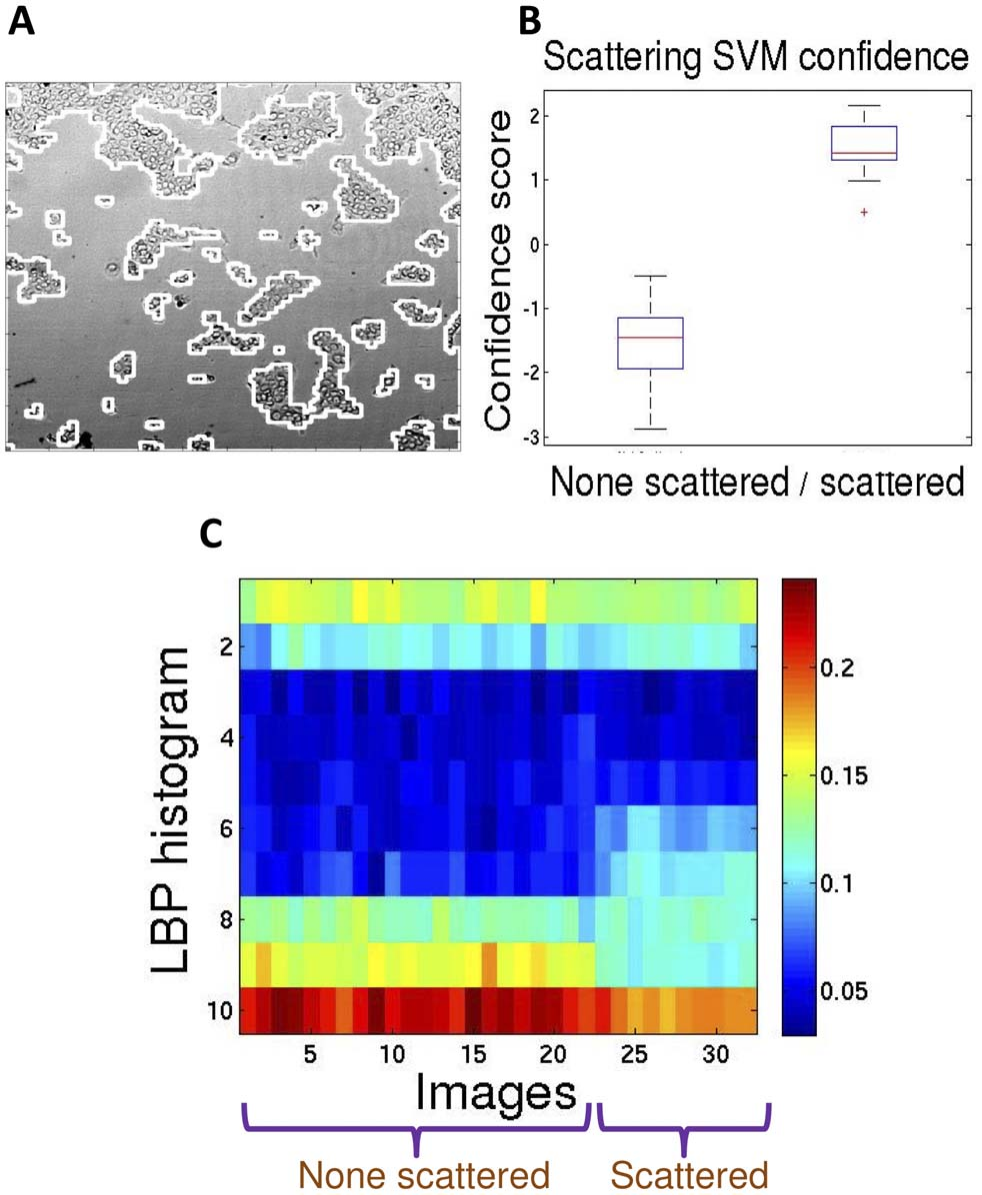
Application to scatter assay automatic classification. (A) An example of MultiCellSeg's performance on cell scattering example image. (B) SVM confidence on scattered/non scattered classification. 100% accurate classification is achieved on the 32 images (p<0.0001 via Wilcoxon rank sum test) both in leave-one-out cross validation and in repeatedly partitioning the data to equal sized train- and test-set, train a SVM on the training set and evaluate on the test set. (C) Visualization of the images' feature space. Each column represents an image's LBP descriptor vector, which is a normalized histogram. The first 22 images are none scattered images, while the last 10 are scattered.

The translation of a given scattering image to a feature vector was performed in the following manner:

- Down-sample the image's spatial resolution such that each pixel is of size 5×5 µm;
- MultiCellSeg was applied to partition the image to cellular and background regions (Fig. 7A); designated classifiers were trained on 4 scatter images for that purpose;
- Local Binary Pattern (LBP) descriptor [29], a gray-scale invariant texture measure for the local-texture of the patch, was extracted for every pixel classified as “cellular”;
- The final descriptor is the normalized histogram of LBP values in all cellular regions.

Evaluation of the proposed cell scattering measure succeeded in all 32 images obtained. The confidence scores of scattered and non-scattered experiments are shown in Fig. 7B: the confidence value zero (the default) is a perfect classifier for this dataset (p<0.0001, Wilcoxon rank sum test), the confidence scores for each image was achieved using “leave one out” cross-validation, as described above. In this case, it is easy to see that the features of the extracted images' texture are highly discriminative (Fig. 7C), and thus it is not surprising that the classifier works so well. As an additional validation step, we partitioned the images to equal size train- and test-sets, where the only constraint was to have more than 3 scattered images in the training set. An SVM was trained on the training set and evaluated on the test set. This process was repeated 100 times, each time selecting independently the training set, and in all executions the classification was perfect with respect to the experts' manual visual classification.

It can be visually observed in Fig. 7C that the most prominent features in the LBP histogram are 6, 7 and 9. This was validated by performing the same analysis on these 3 features and showing that the classification accuracy is still 100% (Fig. S1). However, since LBP is a well-known general texture descriptor we decided to use it as is in the scattering application. For other cell lines or different imaging conditions, the complete histogram might be a more robust descriptor.

## Discussion

Automatic processing of microscopic images is a critical component in analysis of many biological experiments. In recent years, much effort was devoted to develop algorithms and automatic tools for this task. However, most algorithms are designed for fluorescence microscopy, thus bright-field microscopy, which demonstrates morphological alteration and is harder to process, has been neglected.

We suggest a new approach for multi-cellular analysis of bright field microscopy. The main idea is to use the natural textural information for both image segmentation and appearance-based classification tasks. MultiCellSeg is applied on DIC images from time-lapse wound healing experiments to verify that HGF/SF accelerates healing, and to demonstrate that the healing rate is linear both for treated and untreated cells. It is also applied as the first step in a texture-classification application to measure cell scattering, an approach that proved to be extremely accurate, achieving perfect agreement with manual expert's visual tagging.

MultiCellSeg applies classification to the task of image segmentation to cellular and background regions. To the best of our knowledge, this is the first attempt to apply Machine Learning to this problem and to conduct a comprehensive comparison of its performance with that of a segmentation algorithm designed for this purpose. Our approach surpasses the existing algorithms in performing this task for a wide range of scales, illumination conditions, and cell types without the need to tune parameters, which is critical in such applications.

MultiCellSeg's local-patches classification approach significantly surpasses TScratch's in all data sets but one (tscratch, see Fig. 3D). This data set was taken from the TScratch package, with many images that contain scattered cells. The region-classification is designed to deal with this problem. When considering the final segmentation, MultiCellSeg significantly tops the alternative in all data sets.

In principle, the second phase of MultiCellSeg may be plugged in to enhance the performance of Scratch's second phase, but TScratch seems to be less sensitive to small details, which results in significantly less fine regions then with our approach.

Utilization of several types of features on several scales makes MultiCellSeg robust for varying conditions. In contrast to other approaches that tend to refrain from fine details to avoid gross mistakes or use data-specific assumptions, our algorithm operates in higher spatial resolution, detects small regions of interest and then decides whether to keep or to discard them via post processing (regional classification), in a fully automated manner. As a result, in many images where the wound is almost healed, our algorithm performs satisfactorily, whereas other algorithms fail to mark open regions, as exemplified in Fig. 4.

To further enhance the proposed segmentation performance, one can suit a model to fit a specific experiment, cell type or imaging conditions. This can be exceedingly useful nowadays, when high-throughput experiments are performed, each with hundreds of images [13]. To this end, one (or more) image(s) should be manually marked to apply the training phase in our algorithm. This process is only partly automatic, but it requires no-parameter setting and may result in notable improvement in performance with minimal effort.

The automatic, accurate zero-parameters MultiCellSeg may serve as a tool for various biological analyses. MultiCellSeg's Matlab source code is freely available as standalone software to allow others to use it for wound healing analyses, multi-cellular bright field cells segmentation, and for other applications yet to evolve. The source code and accompanying graphical user nterface (GUI) can be found at http://www.cs.tau.ac.il/~assafzar/MultiCellSeg.zip, it is recommended to read carefully the README file (http://www.cs.tau.ac.il/~assafzar/MultiCellSeg_README) before applying it. In the future, we plan to add training capabilities to enable specific designated models for different cell lines and imaging conditions and/or to integrate it as part of a larger project (e.g., [5], [6]).

Wound healing assay is common and is applied by many research groups, but its analysis is very narrow in the sense that only a few measures are considered: the healing rate is calculated over a short period of time. The approach presented here can become the cornerstone for novel methods to be exploited in wound healing analysis. To analyze large data sets such as frequently sampled wound healing assays (acquired by time lapse microscopy), we suggest to perform manual marking of a few images to train a classifier that will be used to segment the entire time-lapse experiment. Producing these high-temporal-resolution progress graphs may reveal biological processes that are currently unknown, such as the linearity of the healing process, as described here.

Another potential corollary is to model the motion patterns of cells throughout the healing process. This is an open question of current interest (e.g., [30], [31], [32]). Modeling cellular motility patterns under stimulants/inhibitors treatments may facilitate the understanding of cell motility mechanisms and enable the development of new anti-metastatic drugs. A correct, high-throughput, partitioning to occupied and background regions can be the first step in developing such an analysis.

An additional application for bright field multi-cellular segmentation is in cell scatter assay. Image texture histogram of cellular regions is used to define the degree of scattering in an inherently different approach than prior attempts that focus on counting single and clustered cells. Information extracted from multi-cellular bright field microscopy can thus be used to distinguish between different molecular-related cellular motility and morphology phenomena.

## Supporting Information

Figure S1 **LBP features 6, 7 and 9 for scatter assay classification.** (A) The most prominent features in the LBP histogram are 6, 7 and 9. Each column represents an image's 6th, 7th and 9th LBP descriptor value. The first 22 images are none scattered images, while the last 10 are scattered. (B) SVM confidence on scattered/non scattered classification based on these 3 features. 100% accurate classification is achieved on the 32 images (p<0.036 via Wilcoxon rank sum test) both in leave-one-out cross validation and in repeatedly partitioning the data to equal sized train- and test-set, train an SVM on the training set and evaluate on the test set.
(TIF)

File S1 **Supporting information text.** Detailed description of the MultiCellSeg algorithm, Receiver Operating Characteristic (ROC curve).
(DOCX)
