# Supplementary figures and images for: Cell Motility Dynamics: A Novel Segmentation Algorithm to Quantify Multi-Cellular Bright Field Microscopy Images
Source: PLoS One. 2011 Nov 9;6(11):e27593. doi: 10.1371/journal.pone.0027593 (PMC3212570; doi:10.1371/journal.pone.0027593)

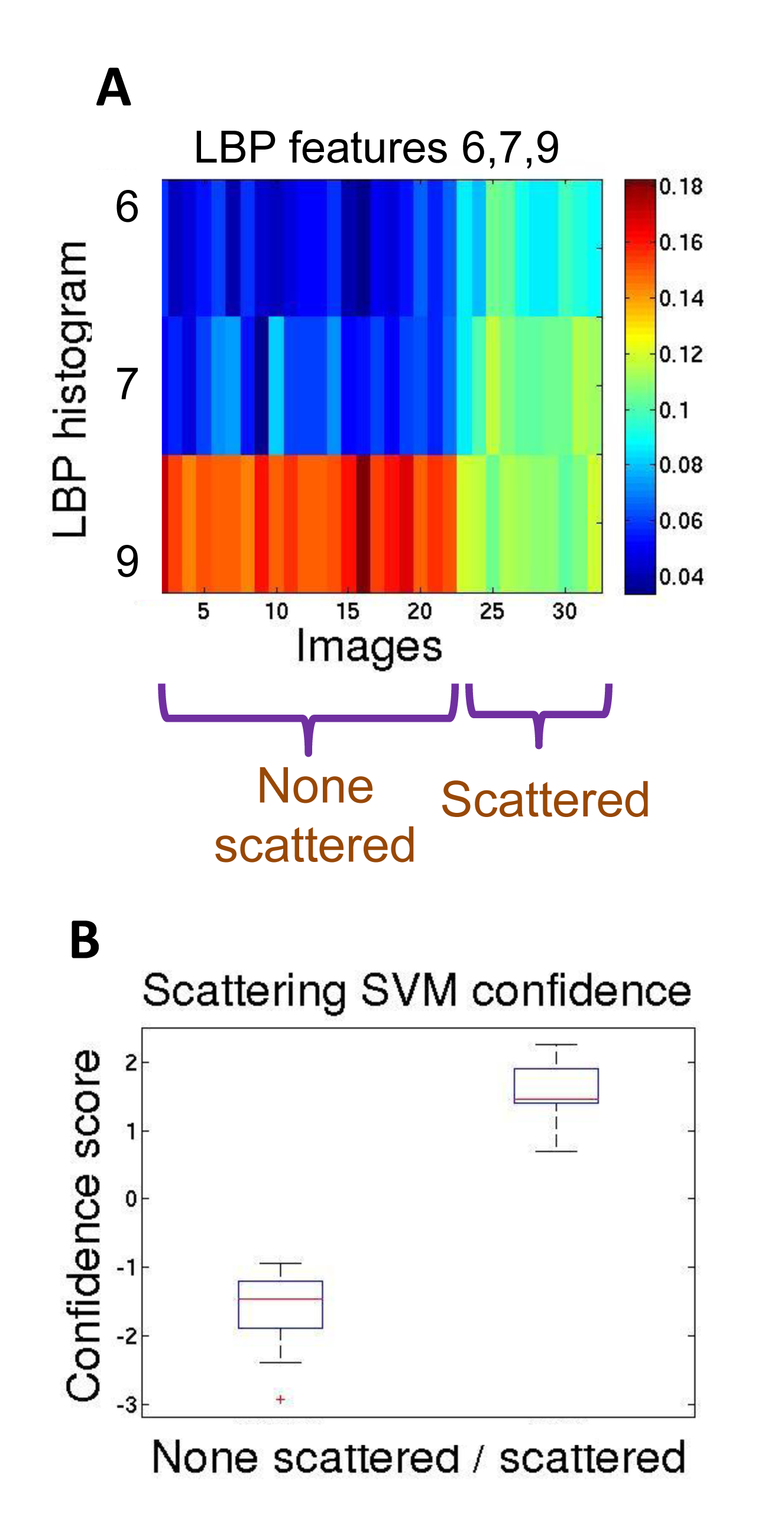

Supplement: Figure S1 — LBP features 6, 7 and 9 for scatter assay classification. (A) The most prominent features in the LBP histogram are 6, 7 and 9. Each column represents an image's 6th, 7th and 9th LBP descriptor value. The first 22 images are none scattered images, while the last 10 are scattered. (B) SVM confidence on scattered/non scattered classification based on these 3 features. 100% accurate classification is achieved on the 32 images (p<0.036 via Wilcoxon rank sum test) both in leave-one-out cross validation and in repeatedly partitioning the data to equal sized train- and test-set, train an SVM on the training set and evaluate on the test set. (TIF) [file pone.0027593.s001.tif]
