# Supplementary material for: Cell Motility Dynamics: A Novel Segmentation Algorithm to Quantify Multi-Cellular Bright Field Microscopy Images
Source: PLoS One. 2011 Nov 9;6(11):e27593. doi: 10.1371/journal.pone.0027593 (PMC3212570; doi:10.1371/journal.pone.0027593)
Supplement: File S1 — Supporting information text. Detailed description of the MultiCellSeg algorithm, Receiver Operating Characteristic (ROC curve). (DOCX) [file pone.0027593.s002.docx]

**Supporting Information**

***MultiCellSeg*: The Segmentation Algorithm**

As described in the main text, the segmentation algorithm consists of a training stage that computes a linear model (classifier) designated to distinguish between background and cellular local patches (*patch classifier*). An additional classifier is trained on spatial clusters of local patches (regions) to reclassify cellular regions that were tagged as “background” by the patches classifier (*region classifier*).

Given an image at test, a grid of patches is overlaid on it; each patch is assigned to the patches classifier to produce the initial segmentation. Next, regions marked as background are reclassified by the regions classifier. Finally, graph-cut segmentation is applied to refine the wound’s contours.

The process is visualized in Fig. 1 in the main text, and the algorithmic details are described in the following paragraphs.

*Learning background patches*

Every image is partitioned into a grid of non-overlapping 20x20 pixels patches. The input for the training set includes the images and their corresponding pixel-wise cell-background baseline marking, performed manually by an expert’s visual inspection. From each patch, five sets of features (or combination of features) are extracted; each describes the patch's statistics to be used for separating cellular patches from background patches, as follows:

- Image (global) and patch (local) gray-level mean and standard deviation;
- Patch's gray level histogram;
- Histogram of patch's gradient intensity, a measure for the amount of details in the patch;
- Histogram of patch's spatial smoothness: the absolute difference between each pixel's intensity and its neighborhood.
- Concatenation of features from a broader area surrounding the patch, including mean and standard deviation of gray-level, gradient intensity and smoothness, as well as gray-level histogram.

Five Support Vector Machines (SVM) [[1](#_ENREF_1)] (from the LIBSVM library [[2](#_ENREF_2)]) are trained, one for each set of features. The output of each SVM is a *confidence score* for every patch that measures the classifier's "certainty" in its classification. The confidence of a given patch is defined as the Euclidian distance of its feature-vector representation to the hyper plane defined by the linear model. The five confidence scores per patch, one from every SVM, are further fed, as a vector of length 5, into an additional training session that calculates the final SVM classifier^[[1]](#footnote-2)^, which is used to put weights on the five classifiers' output to achieve the final classification. Such a combination of the output of multiple classiﬁers is referred to in the literature as stacking [[3](#_ENREF_3)]. It is important to note that the same training set is used to train all six classifiers (five classifiers and the combination of their outputs). The patches-learning phase is illustrated in Fig. 2 in the main text.

To evaluate the contribution of each feature set to the overall patch classifier performance the following analyses were performed:

- Each of the 5 features vectors was evaluated separately in comparison to the combined patch classifier (i.e., using a single feature vector and the corresponding SVM classifier as the patch classifier) on every data set.
- The results obtained using the intensity histogram was significantly inferior to the combined classifier, while the results obtained using the other feature sets were marginally inferior to the features combination.
- Discarding the intensity histogram feature set and training a patch classifier based on the 4 remaining feature sets resulted with inferior performance compared to the classifier that was trained using all 5 feature sets.
- Since the ultimate purpose of the algorithm is to produce accurate and generally robust segmentations, the combination of all 5 feature sets, producing the most accurate segmentation was used.

*Learning cellular regions*

After the classification of local patches, an additional classification of each spatially- connected component of background patches is performed to decide whether it is actually part of the background or not. An additional SVM classifier is trained to perform the global regional classification task. The original image and the patches' confidence map are used to extract the following features per candidate region: the region's size (number of pixels), a histogram based on the original image intensities and another based on the patches' confidence scores. The training session results in a regional classification model later used to discard cells that are tagged as background by the patches' classifier. An example is shown in Fig. 1D in the text.

*Graph-cut segmentation*

The final step is refining the contours to fit the image's gradients map, which represent the true cellular-background edges in the original image. This allows achieving higher spatial resolution in segmentation by using the original image's resolution rather than the patch-size resolution that was enforced by the algorithm until this stage. To this end, the graph-cut segmentation approach [[4](#_ENREF_4),[5](#_ENREF_5),[6](#_ENREF_6)] is applied, the implementation of which is described by Bagon et. al. [[7](#_ENREF_7)]. As input, the algorithm receives a mask that represents the prior knowledge about the image and an energy term that defines the cost of different tagging of neighboring pixels.

Eroded versions of the cellular and the background regions together define the prior mask, and the image gradient is used to specify the energy term. A maximal cut is calculated using these constraints, and the result is defined as the final MultiCellSeg segmentation. An example is illustrated in Figs. 1E-F and 4A-B in the text.

**Receiver Operating Characteristic (ROC curve)**

For ROC analysis true-positives were defined as the background pixels classified correctly as "background", and false-positives were defined as the cellular pixels misclassified as "background". The true-positive rate (TPR) is the percentage of background pixels classified correctly ("background") out of all image's background pixels. This is averaged for all images of the data set. The false-positive rate (FPR) is the percent of cellular pixels classified as "background" out of all image's cellular pixels, also averaged over all images of the data set. The graphs in Fig. 3 were produced by applying a dense-sequence of ongoing thresholds on the confidence scores retrieved from the patches (local) classifier. Each threshold defines a distinct patches-classification, thus a new "point" on the graph of FPR vs. TPR.

**References**

1. Cortes C, Vapnik V (1995) Support-Vector Networks. Machine Learning 20: 273-297.

2. Chang C-C, Lin C-J (2001) LIBSVM : a library for support vector machines.

3. Wolpert DH (1992) Stacked Generalization. Neural Networks 5: 241-259.

4. Boykov Y, Veksler O, Zabih R (2001) Fast approximate energy minimization via graph cuts. Ieee Transactions on Pattern Analysis and Machine Intelligence 23: 1222-1239.

5. Boykov Y, Kolmogorov V (2004) An experimental comparison of min-cut/max-flow algorithms for energy minimization in vision. Ieee Transactions on Pattern Analysis and Machine Intelligence 26: 1124-1137.

6. Kolmogorov V, Zabih R (2004) What energy functions can be minimized via graph cuts? Ieee Transactions on Pattern Analysis and Machine Intelligence 26: 147-159.

7. Bagon S (2006) Matlab wrapper for Graph Cut.

1. Although each of the five feature sets is represented by a vector of different lengths, the SVM is not biased since each set's classifier outputs a single feature that is utilized in the additional training session. [↑](#footnote-ref-2)
